# Processing of the 3C/D Region of the Deformed Wing Virus (DWV)

**DOI:** 10.3390/v15122344

**Published:** 2023-11-29

**Authors:** Carina Maria Reuscher, Sandra Barth, Fiona Gockel, Anette Netsch, Kerstin Seitz, Till Rümenapf, Benjamin Lamp

**Affiliations:** 1 Institute of Virology, Faculty of Veterinary Medicine, Justus-Liebig-University, Biomedical Research Center (BFS), Schubertstrasse 81, 35392 Giessen, Germanysandra.barth-2@vetmed.uni-giessen.de (S.B.); fiona.gockel@bio.uni-giessen.de (F.G.);; 2 Institute of Medical Virology, Justus Liebig University, Biomedical Research Center (BFS), Schubertstrasse 81, 35392 Giessen, Germany; 3 Department for Pathobiology, Institute of Virology, University of Veterinary Medicine, Veterinaerplatz 1, 1210 Vienna, Austria; kerstin.seitz@vetmeduni.ac.at (K.S.); till.ruemenapf@vetmeduni.ac.at (T.R.)

**Keywords:** *Apis mellifera*, deformed wing virus, DWV, polyprotein processing, 3C-like protease, 3CL, RNA-dependent RNA polymerase, RdRp, 3DL, Edman degradation, molecular clone

## Abstract

The deformed wing virus (DWV) belongs to the genus *Iflavirus* and the family *Iflaviridae* within the order *Picornavirales*. It is an important pathogen of the Western honey bee, *Apis mellifera*, causing major losses among honey bee colonies in association with the ectoparasitic mite *Varroa destructor.* Although DWV is one of the best-studied insect viruses, the mechanisms of viral replication and polyprotein processing have been poorly studied in the past. We investigated the processing of the protease-polymerase region at the C-terminus of the polyprotein in more detail using recombinant expression, novel serological reagents, and virus clone mutagenesis. Edman degradation of purified maturated polypeptides uncovered the C- and N-termini of the mature 3C-like (3CL) protease and RNA-dependent RNA polymerase (3DL, RdRp), respectively. Autocatalytic processing of the recombinant DWV 3CL protease occurred at P1 Q_2118_ and P1′ G_2119_ (KPQ/GST) as well as P1 Q_2393_ and P1′ S_2394_ (HAQ/SPS) cleavage sites. New monoclonal antibodies (Mab) detected the mature 3CL protease with an apparent molecular mass of 32 kDa, mature 3DL with an apparent molecular mass of 55 kDa as well as a dominant 3CDL precursor of 90 kDa in DWV infected honey bee pupae. The observed pattern corresponds well to data obtained via recombinant expression and N-terminal sequencing. Finally, we were able to show that 3CL protease activity and availability of the specific protease cleavage sites are essential for viral replication, protein synthesis, and establishment of infection using our molecular clone of DWV-A.

## 1. Introduction

The Western honey bee, *Apis mellifera* (Linnaeus, 1758), is an important pollinator in agriculture, but also in natural ecosystems worldwide [1,2]. Due to its pollination of crops and associated honey production, this honey bee species has been spread around the world and has become irreplaceable in some agricultural fields [3]. The decline in honey bee health observed in recent decades and the recurring winter losses of honey bee colonies in the northern hemisphere are of concern to the wider public [4,5,6]. A combination of several different factors is likely to be the reason for these health problems that have practically wiped out the wild populations of honey bees in northern Europe and North America [7]. In addition to abiotic factors such as global warming [8], intensification of agriculture, industrial-scale management practices [9], and the agricultural use of pesticides [10], multiple biotic factors play a major role for the well-being of honey bees. Recent studies have shown that parasites and viral pathogens are of outstanding importance for the health status and survival of honey bee colonies [11]. Following the emergence of the ectoparasite mite *Varroa destructor* (Anderson and Trueman, 2000) [12], the deformed wing virus (DWV) evolved from a minor pathogen into a serious pest in beekeeping [13]. Varroa mites feed on the fat body of adult honey bees and their capped brood, weakening the host animals and acting as potent vectors for DWV [14,15]. The mite bites penetrate the natural barriers of the bee body and inject the virus directly into the hemolymph. DWV infections are usually asymptomatic in adult bees but cause severe disease in the developing brood, resulting in malformation, dwarfism, discoloration, and ultimately death [16,17]. However, virus infections have different consequences for the bee colony depending on the number of infected individuals and the severity of disease. So-called covert infections, in which the virus is already detectable in single individuals via molecular diagnostics but no clinical signs are yet observed at a colony level, occur regularly throughout the year [18]. If the mite infestation rate increases during the summer, so-called overt infections may result. Overt infections are characterized by the appearance of crippled young bees and eventually by the demise of the whole colony [19]. The outcome of DWV infections depends on different factors, such as the virulence of the respective viral strains [20], the genetic characteristics of the honey bees and their immunological status [21], and ecological factors that the colony encounters. It must be emphasized that the burden of varroa mites, which act as a DWV vector, is certainly the most important factor in the development of overt disease and DWV-related colony collapses [22].

DWV is a member of the genus *Iflavirus* and the family *Iflaviridae* within the order *Picornavirales* [23]. Four master variants of the virus have been described: DWV-A, -B, -C, and -D [24]. Like other members of *Picornavirales*, DWV forms non-enveloped icosahedral virions, in which the RNA is encapsidated by the structural proteins VP1, VP2, and VP3 [25]. The non-segmented single-stranded positive-sensed RNA genome has a length of about 10 kb. It harbors a single open reading frame (ORF) encoding one large polyprotein of 2893 amino acids, which is co- and post-translationally processed into the mature proteins [26]. The initiation of the cap-independent translation is mediated by an IRES element located within the large 5’-UTR of 1167 bps [27]. While the structural proteins are located at the N-terminal half, the non-structural proteins are located at the C-terminus of the polyprotein. A rather short 3´-UTR with a total length of 308 bps is followed by an mRNA-like poly-A tail. Although DWV is one of the best-studied insect viruses, many aspects of the viral lifecycle are still unknown, such as cellular receptors, host factors involved in RNA replication, and last but not least, the exact processing of the viral polyprotein [28].

The processing of the polyprotein is mainly mediated by one major protease in the picornavirus-like iflaviruses. This cysteine protease has been designated a 3C in the viruses within the family *Picornaviridae* [29,30]. It displays a highly conserved catalytic triad consisting of a cysteine, histidine and glutamate, or aspartate residue. The activity of the 3C protease is critical for the processing of the viral polyprotein and is therefore essential for viral replication and genome encapsidation [31]. In addition to the essential cleavage of viral polypeptides, the different 3C proteases also cleave multiple cellular target proteins to manipulate the host cell. Known cellular targets of 3Cs include factors involved in transcription, translation, and nucleocytoplasmic trafficking [32]. The 3C thus modulates metabolism and, most importantly, the cellular mRNA transcription and cap-mediated translation to prioritize cap-independent translation of viral genomes via IRES elements [33,34]. Unprocessed precursor molecules consisting of the 3C protease and the RNA-dependent RNA polymerase (RdRp, 3D) were shown to be required for the initiation of RNA synthesis and are also proteolytically active [35,36]. Based on amino acid sequence alignments and striking secondary structure similarities, comparable 3C-like (3CL) cysteine proteases have been found in all *Picornavirales*, including the iflaviruses [37].

Several studies have already provided insights into the properties of 3CL proteases of iflaviruses. As early as 2012, an active 3CL protease of the Ectropis obliqua virus (EoV) was produced recombinantly, which allowed for the identification of the N- and C-termini of the mature enzyme using autoproteolysis and Edman degradation [37]. The cleavage site nomenclature designates the carboxyterminus generated by the cleavage of a peptide bond as P1, while the newly generated N-terminus is designated P1′ [38]. The identified cleavage sites between P1 Q and P1’ S as well as between P1 E and P1’ A had the typical features of the substrates of many known 3C and 3CL proteases, which usually utilize P1 Q/E and P1’ G/S/A cleavage sites [39]. Ye and colleagues also identified the residues of the catalytic triad within the active center of the EoV protease via an amino acid alignment. They confirmed their prediction via mutational analyses exchanging the amino acids H_2261_, D_2299_, and C_2383_ against alanine residues. While the mutations H_2261_A and C_2383_A completely eradicated the activity of the recombinant enzyme, the mutation D_2299_A resulted in low levels of residual activity. Different cis- and trans-cleavage assays using purified recombinant EoV 3CL protease uncovered basic biochemical properties of the 3CL protease of *Iflaviridae* in vitro. Tests of known serine or cysteine protease inhibitors showed that the 3CL protease of EoV is sensitive to p-chloromercuribenzenesulfonate (PCMBS), methyl methanethiosulfonate (MMTS), and N-ethylmaleimide (NEM), as expected for a cysteine protease. Taken together, the authors were able to provide a complete characterization of the EoV 3CL protease.

In the initial description of DWV, Lanzi et al. identified two potential N-terminal cleavage sites for the 3CL protease with P1 Q_2118_ and P1’ G_2119_ as well as P1 E_2180_ and P1’ G_2181_. They also described potential active amino acid residues of the catalytic triad, namely C_2307_, H_2190_ and D_2225_. Recently, the first enzymatic properties have been reported for recombinant DWV’s 3CL protease (position 2119–2409 of the DWV polyprotein), which specifically cleaved the peptide linking the DWV leader protein-VP2 interface [40]. The N-terminus of DWV 3CL protease, which was already predicted in 2006 [26], was confirmed experimentally using a bacterial expression system approach [40,41]. After the Q_2118_ of the potential N-terminal cleavage site was replaced with an alanine, no maturation at the site was observed. The catalytic triad of the DWV 3CL protease was more accurately predicted using a structural model calculated using AlphaFold2 in combination with further sequence alignments. Alanine substitution experiments indicated that the identity of amino acids H_2170_, C_2307_, and N_2227_ is pivotal for enzyme activity, while D_2225_ is of minor importance. Another study used a very similar system with a short recombinant DWV 3CL protease fragment and a fluorogenic substrate to further determine the optimum temperature of the enzyme [41].

Here, we used an *E. coli*-based expression system to identify the mature products of the processing between the 3CL protease and the 3D-like polymerase (3DL) of DWV. We also generated monoclonal antibodies against the 3CL protease and 3DL to identify the mature proteins and precursors in DWV-infected honey bee pupae. These observations were verified using our molecular clone of DWV-A, demonstrating that the 3CL protease protease activity and the identity of the N-terminal and C-terminal cleavage sites is pivotal for DWV replication in vivo.

## 2. Materials and Methods

### 2.1. Viruses and Honey Bees

Honey bee pupae (*Apis mellifera carnica*) were collected from the apiary of the Institute of Virology, Justus-Liebig University (Giessen, Hesse, Germany) with the permission of the university. Worker bee pupae of the same age were taken from a single comb, which had been freshly introduced in the colony and subsequently labelled after egg deposition to calculate the average age of pupae. Using sterile tweezers, we extracted 13- to 15-day-old pupae with purple to blue eyes from capped brood cells, transferred them into individual wells of 24-well tissue culture plates, and incubated them at 33 °C in a humidified incubator. Damaged or dead individuals were sorted out the next day to achieve uniform experimental conditions. Virus stocks and mock control bees were checked for contamination from other viruses as described previously [42].

Our molecular clone of the DWV-A strain 1414 (GenBank KU847397)) and the reverse genetics system was presented earlier [16]. Briefly, the plasmid DNA of the molecular clone was prepared (Genopure Plasmid Midi Kit; Roche, Basel, Switzerland) and linearized with the restriction enzyme NotI (NEB, Ipswich, MA, USA). Cleavage yielded linear DNA with an SP6 polymerase promoter upstream of the genomic DWV cDNA, which had been cut just downstream of the poly-A tail sequence. Subsequently, the linearized plasmid DNA was purified (Monarch PCR & DNA Cleanup Kit; NEB) and quantified to serve as a template for in vitro RNA transcription (HiScribe SP6 RNA Synthesis Kit; NEB). The RNA was also purified, quantified, and diluted to a final concentration of 1 µg/µL. The bee pupae were transfected with 1 µg of the synthetic RNA by injecting the RNA solution into the thorax segment. Pupae from the same comb were injected with 1 µL of phosphate-buffered saline solution (PBS) to serve as mock controls. All pupae were incubated for 3 days at 33 °C. After the incubation, the bee pupae were frozen at −20 °C, thawed, and then homogenized in 500 µL PBS containing 0.5% Trition X-100. Uniform homogenization was achieved by shaking three times at 4000 rpm for 1 min (MagNA Lyser; Roche, Basel, Switzerland) with the help of stainless-steel beads (Qiagen, Hilden, Germany).

### 2.2. SDS-PAGE, Western Blotting, and Edman Degradation

For sodium dodecyl sulfate—polyacrylamide gel electrophoresis (SDS-PAGE), an SDS-containing 6x loading buffer was added to the honey bee pupae lysates, bacterial lysates, or purified proteins. The SDS-PAGE samples were boiled at 95 °C for 5 min and separated in 7.5% polyacrylamide tricine gels. For the analyses, bee pupae were homogenized in the same way, and equal volumes of virus-positive and negative samples were loaded. Although we do not have a Western blot loading control antibody for honey bees, the similar loading of the lanes can be at least partially reconstructed on the basis of similarly strong background bands. The gels were either stained with Coomassie brilliant blue R-250 or transferred onto Western blot membranes (Pall, Pensacola, FL, USA).

Nitrocellulose membranes were blocked in 5% skim milk (Carl Roth, Karlsruhe, Germany) PBS with 0.1% Tween-20 (Invitrogen, Karlsruhe, Germany). Primary murine monoclonal antibodies (Mabs) were used for protein detection as indicated. Antibody binding was visualized using secondary goat-anti-mouse HRPO conjugates (Dianova, Hamburg, Germany) together with ECL-Prime (GE Healthcare, Chicago, IL, USA). Photon emission was recorded and visualized using a digital imaging system (ChemiDoc; Bio-Rad, Feldkirchen, Germany). Mabs against the DWV 3CL protease (Mab 55B20) and 3DL polymerase (Mab 55A10) were generated in this study. Mab VP1A1 against DWV VP1 and Mab 10B6 anti-His were established earlier [16]. The anti-Flag Mab M2 was commercially purchased (Merck, Darmstadt, Germany).

N-terminal protein sequencing was carried out via phenyl isothiocyanate chemistry as developed by Pehr Edman and analyzed using high-performance liquid chromatography [43]. For this purpose, proteins were transferred to polyvinylidene fluoride (PVDF) membranes using borate buffers, stained with Ponceau S solution (Carl Roth), and excised with a razor blade. Automated Edman degradation was performed by a commercial supplier (Proteome factory, Berlin, Germany).

### 2.3. Recombinant Protein Production

**Figure 1:**
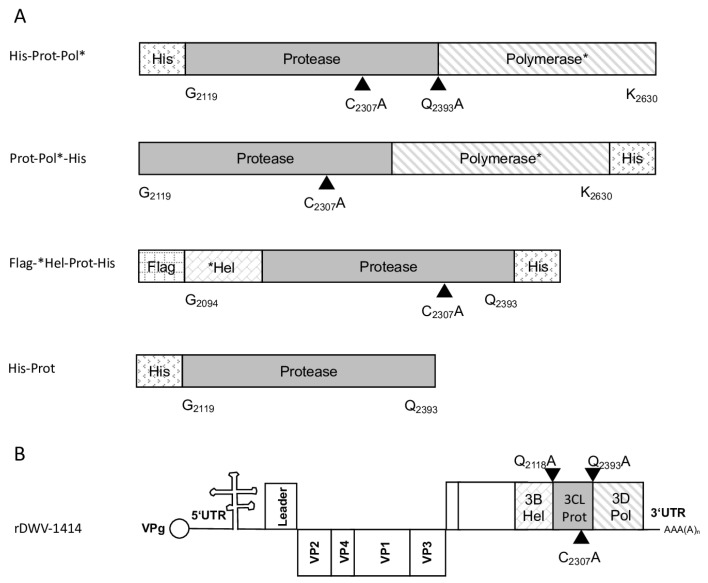
Polyprotein fragments and virus genomes used in this study. (**A**) Scheme of the recombinant proteins used for the determination of 3CL and 3DL cleavage sites and boundaries. Protein tags (His and Flag) were attached to the N- or C-terminus of the viral polyprotein fragments as indicated. The polyprotein positions were specified according to the format of amino acid and residue number (Xn), indicating the boundaries of the recombinant polyprotein fragments. All residue numbers refer to DWV-A strain 1414. Arrowheads indicate the position of cleavage sites and the central cysteine residue of the catalytic triad. An asterisk in designations is to indicate that it is only a fragment of the respective protein. (**B**) Genome organization of DWV-A with annotation of the processing sites characterized in this study.

The molecular clone of the DWV-A strain 1414 served as a DNA template to amplify the 3CL protease and parts of the 3DL gene. The ORF region encoding amino acids G_2119_-K_2630_ was amplified via PCR (Phusion High-Fidelity DNA Polymerase; NEB) using oligonucleotides 5′-3C-Prot_fwd and Internal-Pol_rev. The sequences of the oligonucleotides used in this study are listed in Table 1. A suitable vector backbone of a bacterial expression plasmid (pet11a; Merck KGaA, Darmstadt, Germany) containing an N-terminal 10x-His-tag was amplified using the oligonucleotides 5′-3C-overhang-pet11a_rev and Internal-Pol-overhang-pet11a_fwd, adding complementary extensions for DNA recombination. After purification of the respective PCR products, an in vitro DNA assembly reaction (NeBuilder; NEB) yielded the expression vector pet11a-His-Prot-Pol*. The recombinant expression constructs used in this study and a genome scheme of DWV mutations are presented in an overview figure (Figure 1).

The DNA cloning procedure was carried out in non-expression *E. coli* host cells of strain HB101. For expression, purified plasmid DNA was transferred into expression host cells of the *E. coli* strain Rosetta (Merck) containing a chromosomal copy of the T7 RNA polymerase gene under lacUV5 control. Rosetta cells were grown in Luria–Bertani media until they reached an optical density at 600 nm (OD_600_) of 0.7–0.8. Then, IPTG was added to a final concentration of 1 mM to induce expression of T7 RNA polymerase, which in turn transcribed the recombinant protein gene. After an incubation of 4 h at 37 °C, the cells were harvested via centrifugation (4000× *g* for 10 min), and the cell pellet was resuspended in PBS containing 0.5% Triton X-100. Cell lysis was performed using three cycles of freezing and thawing according to the manufacturer’s recommendation. The His-Prot-Pol* protein was completely insoluble after cell lysis—most probably due to inclusion body formation. Hence, the lysate was cleared via centrifugation (10,000× *g* at 4 °C for 30 min), the supernatant was discarded, and the remaining pellet of insoluble material was resolubilized in PBS containing 8 M urea. The resolubilized protein solution was subjected to immobilized metal ion affinity chromatography (IMAC) on an ÄKTA pure 25 device (Cytiva, Marlborough, MA, USA). After binding to Ni-Sepharose (HisTrap HP; Cytiva) and being washed with 8 M urea PBS, the protein was eluted in 8 M urea PBS, using an imidazole gradient (0 to 500 mM). The elution process was monitored via UV absorption (280 nm), and the identity of the eluted protein was verified using SDS-PAGE and subsequent Western blot analysis with Mab 10B6 anti-His. The protein-containing elution fractions were pooled, and urea was diluted out stepwise. The addition of PBS to the denatured samples was sufficient to fold the protein back to a soluble state without recognizable losses. The soluble protein was re-concentrated via ultrafiltration (Amicon Ultra-15, 30 kDa MWCO; Millipore, Burlington, MA, USA) and washed twice to remove last urea contaminants.

### 2.4. Generation of Monoclonal Antibodies

The recombinant His-Prot-Pol* was homogenous and suitable for the immunization of mice, which was carried out by a commercial provider (Davids Biotechnologie GmbH, Regensburg, Germany). After successful immunization of three Balb/c mice, spleen cells of hyperimmune animals were harvested, fused with SP2/0-Ag14 myeloma cells, and selected using the hypoxanthine-aminopterin-thymidine (HAT) method. The supernatants of individual hybridoma cell clones were screened for reactive antibodies via indirect enzyme-linked immunosorbent assay (ELISA) again using the recombinant His-Prot-Pol* as an antigen source. For primary validation of Mab reactivity, we harvested cells of induced and non-induced bacterial cultures expressing His-Prot-Pol*. These cells were lysed, subjected to SDS-PAGE, and blotted onto nitrocellulose membranes. Subsequently, the supernatants of all ELISA-reactive hybridoma clones (more than 100) were tested. To determine whether the individual antibodies were directed against the protease or against the polymerase moiety of our immunogen, another expression plasmid was generated. This plasmid contained only the 3CL protease (G_2119_-Q_2393_) as experimentally determined via Edman degradation and described in the results section. The expression plasmid pet11a-His-Prot was generated by deleting the polymerase sequences from the expression plasmid pet11a-His-Prot-Pol* using a Phusion reaction (NEB) using the oligonucleotides NcoI-3′-3C_rev and NcoI-Stop-pet11a_fwd. The PCR was digested with the enzymes DpnI (NEB), which removed template DNA, and NcoI (NEB), which generated sticky PCR product ends. The product of digestion was purified and ligated using T4-DNA-Ligase (Instant Sticky-end Ligase Master Mix, NEB). Cell lysates from induced Rosetta cultures expressing either His-Prot or His-Prot-Pol* were resolved side-by-side in SDS-PAGE and blotted to analyze to binding of Mabs to the different protein domains.

### 2.5. Analysis of the Processing of Recombinant DWV Polyprotein Fragments

For the determination of the C-terminal cleavage site of the DWV 3CL protease, we C-terminally His-tagged the identical polyprotein fragment consisting of the 3CL protease fused to the stretch of the N-terminus of the polymerase region (Pol*). The pet11a-Prot-Pol*-His vector thus encompassed the polyprotein amino acids G_2119_-K_2630_. A PCR product of this ORF fragment was generated using the oligonucleotides pet11a-overhang-5′-3C_fwd and His-overhang-Internal-Pol_rev. A suitable vector backbone with a C-terminal His-tag for the expression plasmid was amplified via PCR, using oligonucleotides Internal-Pol-overhang-His_fwd and 5′-3C-overhang-pet11a_rev. The PCR products were recombined via DNA assembly.

The 3CL protease was inactivated in a negative control plasmid by introducing the amino acid exchange C_2307_A. Replacement of the active cysteine within the catalytic triad should inactivate the enzyme as shown by Yuan et al. [40], and hence block autocatalytic processing. Oligonucleotides 3C-C_2307_A_fwd and Internal-Beta-Lactamase_rev were used to amplify one fragment of the plasmid, whereas 3C-C_2307_A_rev and Internal-Beta-Lactamase_fwd were used for the other to generate pet11a-ProtC_2307_A-Pol*-His.

The glutamine at position 2393 was changed to an alanine (Q_2393_A) to destroy the determined cleavage site between the protease and the polymerase. For this purpose, two separate fragments were again amplified via PCR, and the mutation was encoded within the primer sequences. Homologous recombination was used to reassemble the fragments using the same strategy with a second assembly site in the beta-lactamase gene, resulting in pet11a-ProtQ_2393_A-Pol*-His.

For the determination of the N-terminal cleavage site of the DWV 3CL protease, we generated another N- and C-terminally tagged polyprotein fragment consisting of an N-terminal Flag-tag, a short stretch from the C-terminus of the helicase (*Hel), the complete 3CL protease and a C-terminal His-tag sequence. The DWV ORF sequence coding amino acids G_2094_–Q_2393_ was amplified using the oligonucleotides Flag-Internal-Hel_fwd and His-3′-3C_rev adding the Flag-tag sequence on the 5′-end and a His-tag sequence at the 3′-end of the PCR product. A suitable backbone of a pet11a expression vector, already containing an N-terminal Flag-tag and a C-terminal 10x His-tag was amplified via PCR using the oligonucleotides ATG-Flag _rev and 3C-overhang-His-tag_fwd. The in vitro assembly reaction generated the expression vector pet11a-Flag-*Hel-Prot-His.

A negative control with the mutation C_2307_A was also generated. For this purpose, we used the oligonucleotides 3C-C_2307_A_fwd and 3C-C_2307_A_rev and again employed the beta-lactamase gene assembly site. The respective expression construct was termed pet11a-Flag-*Hel-ProtC_2307_A-His.

The glutamine at position 2118 was changed to an alanine (Q_2118_A) to destroy the determined cleavage site between the helicase and the protease. For this purpose, two separate fragments were again amplified via PCR and the mutation was encoded within the primer sequences (3C-Q_2118_A_fwd and 3C-Q_2118_A_rev). Homologous recombination was used to reassemble the fragments using the same strategy with a second assembly site in the beta-lactamase gene, resulting in pet11a- Flag-*HelQ_2118_A-Prot-His.

### 2.6. Analysis DWV Polyprotein Processing In Vivo

DNA assembly reactions were used to introduce single amino acid exchanges into the ORF of our molecular clone of DWV using a very similar strategy. The cysteine at position 2307 was exchanged against alanine employing the same set of oligonucleotides using the assembly sites at the target position as well as in the beta-lactamase gene. Only the PCR products became significantly larger because the entire viral genome and the pBR322 vector framework had to be amplified. An assembly of the respective PCR products yielded a plasmid harboring the mutated genome of rDWV-C_2307_A. In a subsequent attempt, the glutamine at position 2118 was exchanged against alanine using oligonucleotides 3C-Q_2118_A_fwd and 3C-Q_2118_A_rev to validate the importance of the cleavage site in the viral infection cycle using clone rDWV-Q_2118_A. Similarly, Q_2393_ was exchanged against an alanine using the oligonucleotides 3C-Q_2393_A_fwd and 3C-Q_2393_A_rev. Consequently, this cleavage site mutant cDNA clone was termed rDWV-Q_2393_A. The histidines at positions 2170 and 2190 were exchanged against alanine employing oligonucleotides 3C-H_2170_A_fwd/_rev and 3C-H_2190_A_fwd/_rev. The aspartates at positions 2199 and 2225 were exchanged against alanine employing oligonucleotides 3C-D_2199_A_fwd/_rev and 3C-D_2225_A_fwd/_rev. And finally, the asparagine at position 2227 was exchanged against an alanine with the oligonucleotides 3C-N_2227_A_fwd/_rev. The resulting virus were named in a consistent scheme as rDWV-X_nnnn_A.

The successful mutagenesis and integrity of the plasmids generated in this study were verified restriction enzyme mapping, initial Sanger sequencing (Microsynth, Balgach, Switzerland), and complete ONT plasmid sequencing (Full PlasmidSeq, Microsynth), which documented error-free cDNA clone sequence. Further details on the DNA cloning and recombination strategies used here will be provided upon request.

## 3. Results

### 3.1. A Polyprotein Fragment Containing the 3CL Protease Undergoes Self-Cleavage

**Figure 2:**
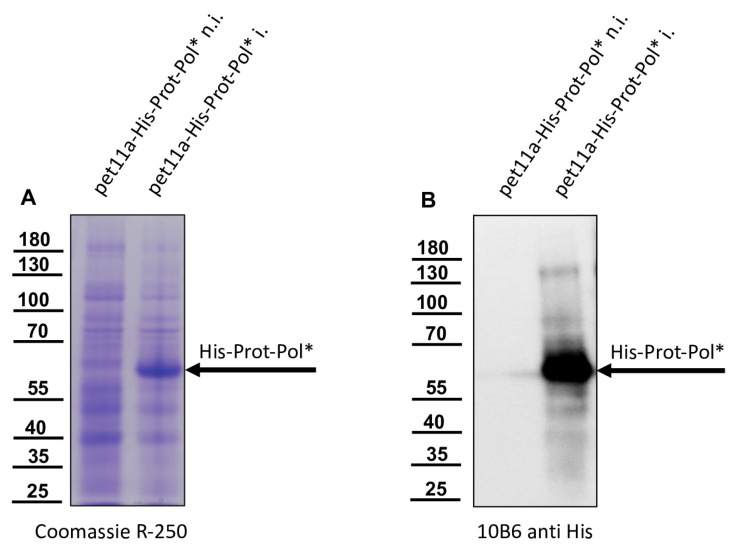
Recombinant expression of DWV His-Prot-Pol*. (**A**) Total *E. coli* protein of non-induced and induced cultures transformed with the plasmid pet11a-His-Prot-Pol* were resolved via SDS-PAGE and visualized via Coomassie stain. Note the overexpression of a protein with an apparent molecular mass of 60 kDa in the induced culture. (**B**) Western blot analysis of total *E. coli* protein of non-induced and induced cultures transformed with the plasmid pet11a-His-Prot-Pol*. Note the reactivity of the anti-His-tag Mab with the overexpressed protein. The recombinant protein is marked with an arrow (His-Prot-Pol*).

We generated a pet11a vector encoding a polyhistidine-tagged polyprotein fragment spanning amino acids G_2119_-K_2630_ of DWV-A (strain 1414, GenBank: KU847397.1) [16]. When the DWV genome was first described, it was already suspected, on the basis of sequence similarities to known 3CL proteases and other similar cleavage sites in the polyprotein, that G_2119_ represents the first amino acid of the mature 3CL protease [26]. Experimental confirmation of a cleavage between P1 Q_2118_ and P1’ G_2119_ was achieved via expression of a recombinant glutathione S-transferases (GST)-Vpg-3C fusion protein and mutation of residue Q_2118_ by Yuan et al. [40]. We named the G_2119_-K_2630_ polyprotein fragment His-Prot-Pol*, assuming that it consists of the entire 3CL protease fused to a polymerase fragment at the C-terminus. This polyprotein fragment was well expressed in *E. coli* (strain Rosetta), as shown via a two-hour test expression at 37 °C, which revealed an overexpressed protein with an apparent molecular mass of 60 kDa in both Coomassie staining (Figure 2A) and anti-His Western blot (Figure 2B) fitting to the calculated molecular mass of His-Prot-Pol* (58.5 kDa).

**Figure 3:**
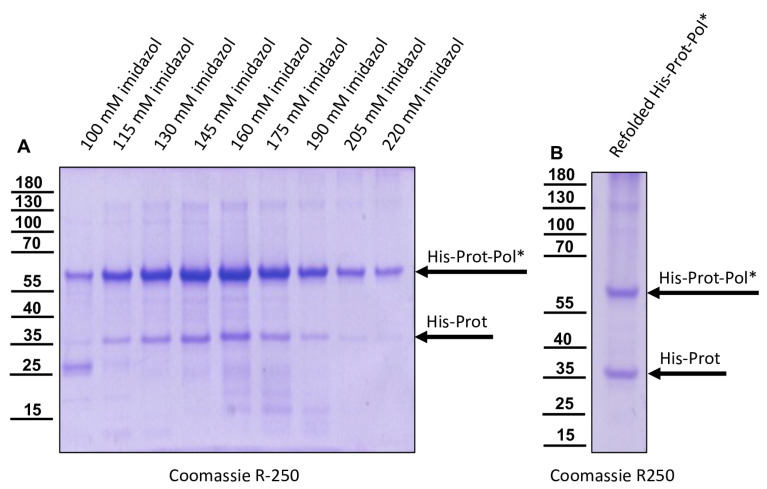
IMAC purification of His-Prot-Pol*. (**A**) The insoluble fraction of a lysate from induced cultures of *E. coli* transformed with the plasmid pet11a-His-Prot-Pol* was solubilized in 8 M urea buffer and subjected to IMAC. The individual imidazole elution fractions were resolved via SDS-PAGE and stained using Coomassie. Note the two different protein species with an apparent molecular mass of 60 kDa (His-Prot-Pol*) and 35 kDa (His-Prot) that were specifically eluted with increasing imidazole concentrations. (**B**) Refolded IMAC purified His-Prot-Pol* and His-Prot after the addition of PBS and re-concentration using an ultrafiltration device. His-Prot-Pol* and His-Prot are marked with an arrow.

No or only minor processing of His-Prot-Pol* was observed in this Western blot, showing no additional bands of the tagged protein. We analyzed the solubility of His-Prot-Pol* after mass production for 6 h at 37 °C and cell lysis in 1% Triton X-100 PBS and detected the protein solely in the insoluble matter. The complete insolubility, likely due to the formation of highly aggregated protein complexes commonly referred to as *E. coli* inclusion bodies, was also observed at other expression temperatures or shorter expression times. We decided against an additional fusion with solubility-mediating proteins like GST, because we wanted to prepare serological reagents against the protein. Hence, we purified His-Prot-Pol* under denaturing conditions from the insoluble matter dissolved in 8 M urea buffer. IMAC and elution with a linear imidazole gradient resulted in a peak release of the His-tagged protein from the Ni^2+^-column at a concentration of 150 mM imidazole (Figure 3A). The still-denatured protein was refolded with 8 M urea buffer using the stepwise addition of PBS. After re-concentration by ultrafiltration, the purity of the soluble protein was determined via SDS-PAGE and subsequent Coomassie staining (Figure 3B).

**Figure 4:**
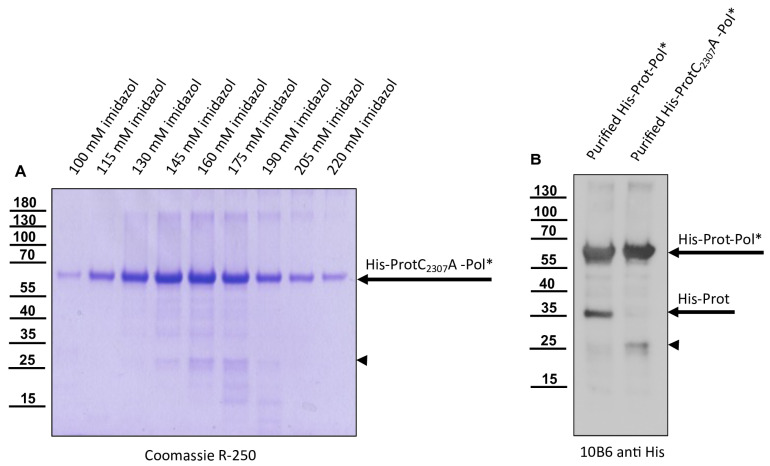
The proteolytically inactive variant His-ProtC_2307_A-Pol* does not release His-Prot. (**A**) The insoluble fraction of a lysate from induced cultures of *E. coli* transformed with the plasmid pet11a-His-ProtC_2307_A-Pol* was solubilized in 8 M urea buffer and subjected to IMAC. The individual imidazole elution fractions were resolved via SDS-PAGE and stained. Note the single protein species with an apparent molecular mass of 60 kDa that specifically eluted with increasing imidazole concentrations and the absence of a 35 kDa band (His-Prot). (**B**) Refolded IMAC-purified His-Prot-Pol* and His-ProtC_2307_A-Pol* were resolved side by side in SDS-PAGE and analyzed via Western blot using a His-tag-specific antibody. His-Prot of 35 kDa is not formed in the case of the inactivated variant His-ProtC_2307_A-Pol*. However, a weak band at 25 kDa (marked with an arrowhead in (**A**) and (**B**)) appeared, probably representing a product of degradation after prolonged expression time.

The prolonged expression time and purification using the N-terminal histidine tag yielded a distinct additional band at approximately 35 kDa (His-Prot) in both the primary denatured elution fractions and the refolded soluble fraction. The refolded protein preparation was incubated at different temperatures and in different buffer conditions for an extended period without showing any further degradation of the 60 kDa protein. However, the 35 kDa protein was considered to be a cleavage product of autoproteolysis. This was controlled using a proteolytically inactive variant, His-ProtC_2307_A-Pol*. Since expression and purification of His-ProtC_2307_A-Pol* using IMAC showed no additional 35 kDa protein fragment (Figure 4), we concluded that the cleavage was mediated exclusively by the activity of the 3CL protease. We decided to generate serological reagents to further characterize and monitor the cleavage event.

### 3.2. Murine Mabs against the Protease-RdRp-Region of DWV

**Figure 5:**
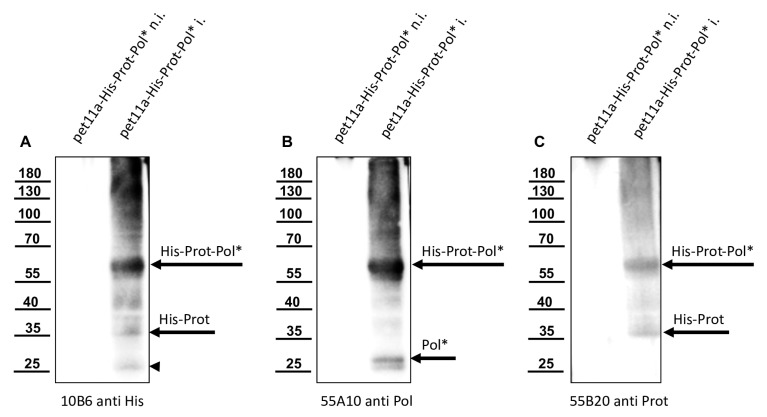
Detection of different products of autoproteolysis of His-Prot-Pol*. Total protein of non-induced (n.i.) and induced (i.) cultures of *E. coli* transformed with plasmid pet11a-His-Prot-Pol* were resolved in SDS-PAGE and analyzed via Western blot. (**A**) Mab 10B6 anti-His showed the full-length His-Prot-Pol* (60 kDa) and the histidine-tagged cleavage product His-Prot (35 kDa). Again, a weak 25 kDa band (marked with an arrowhead) appeared, which was not further characterized. (**B**) Mab 55A10 anti-Pol reacted with the 60 kDa His-Prot-Pol* and two smaller fragments of 26 and 28 kDa representing the released Pol* moiety and an uncharacterized fragment. (**C**) Mab 55B20 anti-Prot in contrast showed a pattern similar to the His-tag specific Mab with bands at 60 and 35 kDa. His-Prot-Pol*, His-Prot, and Pol* fragments are marked with arrows.

We used the re-folded His-Prot-Pol* for the immunization of mice, which was performed at a commercial provider (Davids Biotechnologie GmbH). Antibody-secreting hybridoma cell clones were generated via PEG fusion of SP2/0-Ag14 myeloma cells with freshly prepared or frozen spleenocytes. After HAT selection, cell culture supernatants of individual hybridoma clones were tested in an indirect ELISA using His-Prot-Pol* as a test antigen. The reactivity and specificity of the ELISA-positive Mabs was validated in Western blot analyses using non-induced and induced *E. coli* cultures expressing His-Prot-Pol*. An anti-His antibody (Mab 10B6) control Western blot showed a strong reactivity against the His-Prot-Pol* (60 kDa) in the induced culture, as seen before. Several weaker signals with low molecular mass proteins were simultaneously observed, while no reactivity was observed with the non-induced *E. coli* sample (Figure 5A). Similar results were observed with the newly generated His-Prot-Pol* ELISA-positive antibodies, which showed no signals in the non-induced *E. coli* culture, while strong reactivities were observed with the 60 kDa His-Prot-Pol* (Figure 5B,C). Based on additional weaker reactivities with proteins of lower molecular masses, two specificity patterns could be distinguished among the newly generated His-Prot-Pol*-specific Mabs. One group of Mabs reacted with two additional protein bands with apparent molecular masses of 26 and 28 kDa. The reactivity of Mab 55A10, which exhibited the strongest reactivity of all Mabs generated in the study, is presented as an example for this group (Figure 5B). These antibodies probably reacted with epitopes within the C-terminal part of the His-Prot-Pol* fusion protein, as inferred from the fact that the recognized fragments of 26 and 28 kDa were not detected by the anti-His Mab. The other group of His-Prot-Pol* specific Mabs showed an additional weak signal with a smaller protein (apparent molecular mass of 35 kDa), which was also weakly detected by the His-tag Mab likely representing an N-terminal protease fragment. From this group, Mab 55B20 showed the strongest reactivity against the recombinant fusion protein (Figure 5C).

### 3.3. The N-and C-Termini of the DWV 3CL Protease

**Figure 6:**
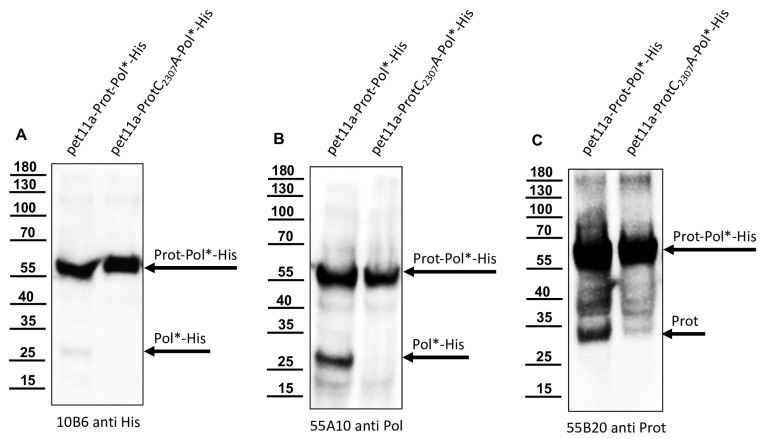
C-terminal histidine-tagged Prot-Pol*-His is also processed by the 3CL protease. Total proteins of induced cultures of *E. coli* transformed with plasmid pet11a-Prot-Pol*-His and pet11a-ProtC_2307_A-Pol*-His were resolved in SDS-PAGE and analyzed via Western blot. (**A**) Mab 10B6 anti-His showed the full-length Prot-Pol*-His (60 kDa) and the histidine-tagged cleavage product Pol*-His with 28 kDa only in the case of the active protease. (**B**) Mab 55A10 anti-Pol reacted with the 60 kDa Prot-Pol*-His and the smaller fragment of 28 kDa representing the released Pol*-His. (**C**) Mab 55B20 anti-Prot showed band at 60 kDa and a free protease of 33 kDa.

His-tag as well as His-Prot-Pol* specific Mabs detected low amounts of distinct smaller protein species, which could be addressed as products of autocatalytic processing steps (Figure 5). We designed vectors for the expression of N- and C-terminally tagged polyprotein fragments, together with appropriate controls with an inactivated 3CL protease. For the determination of the C-terminal cleavage site of the DWV 3CL protease, we shifted the His-tag to the C-terminus of our polyprotein fragment and named the construct Prot-Pol*-His. A proteolytically inactive variant of Prot-Pol*-His was generated via the exchange of active cysteine for alanine (ProtC_2307_A-Pol*-His). Both expression constructs were produced in *E. coli* and subjected to SDS-PAGE side by side. Comparison of the expressed proteins using an anti-His-tag antibody revealed unprocessed Prot-Pol*-His with 60 kDa and a C-terminal product of autoproteolysis with an apparent molecular weight of 28 kDa. The smaller fragment was visible solely in the proteolytically active Prot-Pol*-His and not observed in the ProtC_2307_A-Pol*-His (Figure 6A). The antibody 55A10, which was hypothesized to be directed against the 3DL, showed a picture corresponding to the His-tag antibody (Figure 6B). In contrast, antibody 55B20, which is believed to target the 3CL protease, showed a second protein of approximately 35 kDa in addition to the Prot-Pol*-His corresponding to the mature 3CL protease (Figure 6C).

**Figure 7:**
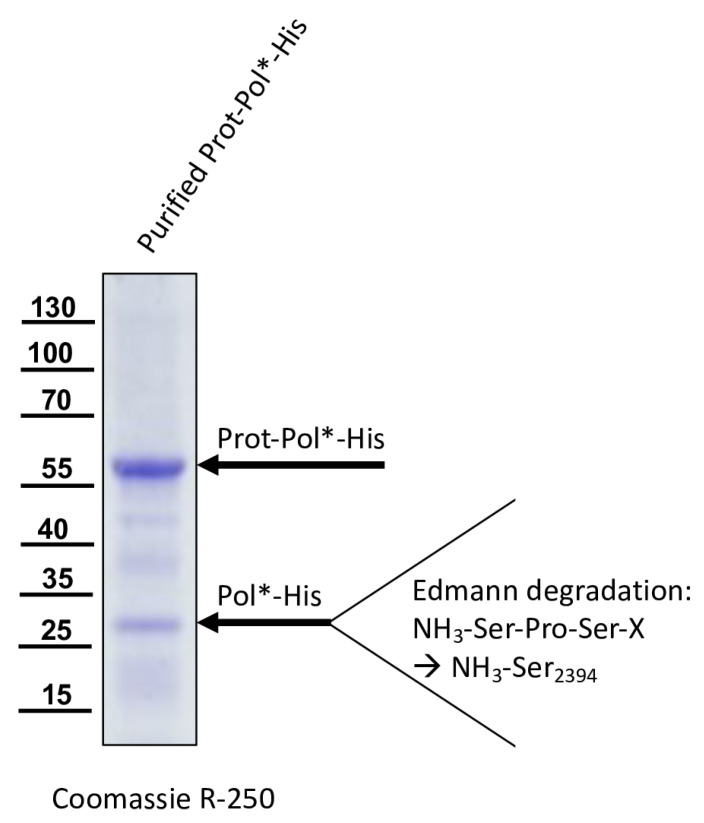
N-terminal sequencing of a purified processed Pol*-His fragment. Prot-Pol*-His was purified via IMAC and subjected to SDS-PAGE. The Coomassie stained gel showed the unprocessed Prot-Pol*-His (60 kDa) and the cleaved Pol*-His fragment (28 kDa). After blotting onto a PVDF membrane and staining, the Pol*-His fragment was excised and analyzed via Edmann degradation. The small amount of protein in the 28 kDa band led to an early termination of the sequencing reaction, and only the first three degradation steps yielded clear results. Since the sequence of the expression construct was known, the amino acid sequence serine (step 1), proline (step 2), and serine (step 3) could nevertheless be assigned to cleavage at position P1′ S_2394_.

After IMAC purification of Prot-Pol*-His, the Pol*-His fragment was blotted onto a PVDF membrane, stained with Ponceau S, excised from the membrane, and subjected to Edmann degradation (Figure 7). Due to the limited quantity of the Pol*-His fragment, the overall signals of the 3-phenyl-2-thiohydantoic acid (PTH) amino acids generated via Edmann degradation were rather weak. Nevertheless, an assignable sequence of amino acids could be determined via degradation steps one to three. Residue P1’ could be identified as serine, P2’ as proline, and P3’ as serine. This sequence indicated cleavage at a P1 Q_2393_ and P1’ S_2394_ cleavage site (Q/SPS), which is only found at one position in the Prot-Pol*-His polypeptide. In addition, the calculated molecular mass of 27.8 kDa and the observed molecular mass of 28 kDa matched well.

**Figure 8:**
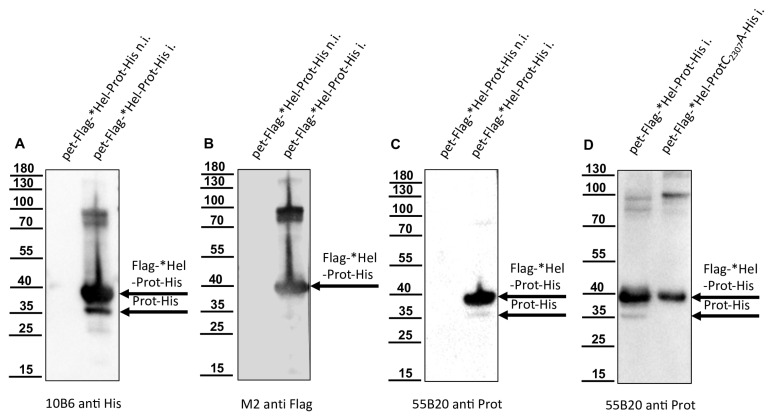
A helicase fragment is autoproteolytically cleaved from the N-terminus of Flag-*Hel-Prot-His. Total protein of non-induced (n.i.) and induced (i.) cultures of *E. coli* transformed with plasmid pet11a-Flag-*Hel-Prot-His were resolved in SDS-PAGE and analyzed via Western blot. (**A**) Mab 10B6 anti-His showed the full-length Flag-*Hel-Prot-His (38 kDa) and the histidine-tagged cleavage product Prot-His with 35 kDa. (**B**) Mab M2 anti-Flag reacted solely with the 38 kDa Flag-*Hel-Prot-His. Note that the N-terminal cleavage product Flag-*Hel with a calculated molecular mass of only 3.8 is not resolved by the 8% acrylamide gel. (**C**) Mab 55B20 anti-Prot in contrast showed a pattern similar to the His-tag specific Mab with a strong band at 38 and a weak band at 35 kDa. (**D**) In direct comparison between induced cultures of proteolytically active Flag-*Hel-Prot-His and Flag-*Hel-ProtC_2307_A-His inactivated by cysteine exchange, the protease specific antibody 55B20 shows that the cleavage between the helicase and protease portion depends on the activity of the protease itself.

Knowing the C-terminus of the mature protease, we aimed to confirm the N-terminus of the mature 3CL protease. For this purpose, amino acids G_2094_-Q_2393_ of the DWV polyprotein were fused with an N-terminal Flag-Tag and a C-terminal His-Tag, resulting in the Flag-*Hel-Prot-His. Western blots showed a strong protein band with an apparent molecular weight of 38 kDa with His-tag, Flag-tag, and protease specific Mabs (Figure 8).

**Figure 9:**
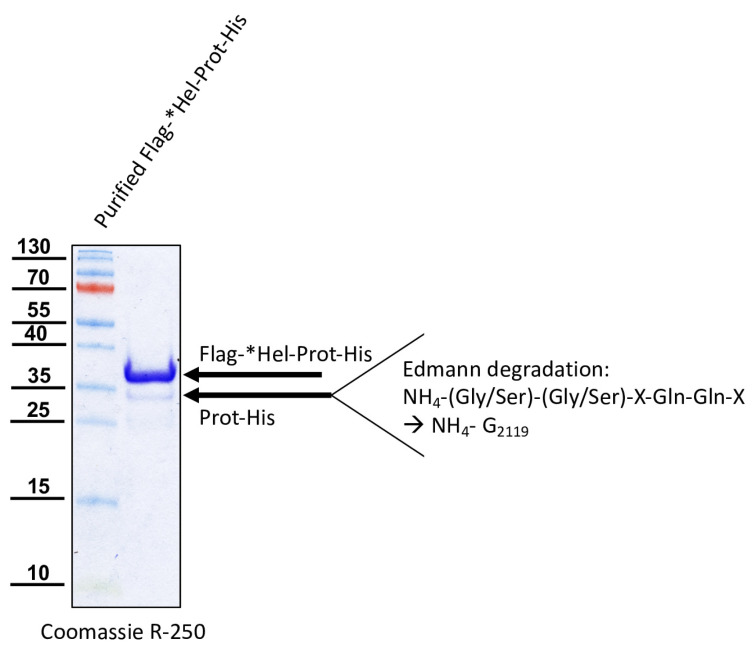
N-terminal sequencing of the purified Prot-His fragment uncovered the N-terminal site of processing. Flag-*Hel-Prot-His was purified via IMAC and subjected to SDS-PAGE. The Coomassie stained gel showed the unprocessed Flag-*Hel-Prot-His (38 kDa) and the cleaved Prot-His fragment (35 kDa). After blotting onto a PVDF membrane and staining, the Pol*-His fragment was excised and analyzed via Edmann degradation. Again, the small amount of protein in the 35 kDa band led to a limited number of evaluable degradation cycles. However, a sequence with amino acids glycine or serine (step 1), glycine or serine (step 2), an unassignable amino acid (step 3), and two glutamines (step 4 and step 5) could be clearly assigned to the cleavage position P1′ G_2119_.

This protein species of 38 kDa represented the unprocessed full-length fusion protein (Flag-*Hel-Prot-His, calculated molecular mass: 35.4 kDa). Another protein band with an apparent molecular weight of about 35 kDa became visible only in the case of the active 3CL protease using the anti-His and the protease specific Mab 55B20 (Figure 8A,C) but was not shown by the anti-Flag antibody (Figure 8B). As a control, we also inactivated the 3CL protease in this construct, introducing the single amino acid exchange C_2307_A. Western blot analysis with our protease-specific antibody of induced *E. coli* cultures expressing Flag-*Hel-Prot-His and the enzymatically inactive variant Flag-*Hel-ProtC_2307_A-His were shown (Figure 8D). We concluded that the Flag-tagged N-terminus of the fusion protein was cleaved off by the activity of the 3CL protease. An N-terminal cleavage product carrying the Flag tag could not be detected in this Western blot analysis due to its very low molecular mass (calculated molecular mass of 3.8 kDa). Alongside the full-length protein Flag-*Hel-Prot-His, the mature protease (Prot-His) was purified using IMAC. After SDS-PAGE and blotting onto a PVDF membrane, the Prot-His was subjected to Edmann degradation (Figure 9). Again, the low amount of cleaved and blotted Prot-His caused low signal intensities for the PTH amino acids generated from the stepwise chemical cleavage. However, Edmann degradation revealed a sequence that could be unambiguously assigned to a single position in the Flag-*Hel-Prot-His polypeptide, because P1’ as well as P2’ could be identified as serine or glycine residues. The results for P3’ were not accurately classifiable and were only indicative of the amino acids S, Q, T, G, or E. However, residues P4’ and P5’ were identically specified as glutamine residues. This sequence matched the already known P1 Q_2118_ and P1′ G_2119_ cleavage site (Q/GSTQQ) not only because of the initial glycine-serine pair, but also because of the two glutamines at position P4’ and P5’, which occur only at this position in the whole polypeptide.

### 3.4. Mab 55B20 Is Reactive against the Mature 3CL Protease, While Mab 55A10 Binds within the RdRp Region

Based on their reactivity against the products of the intramolecular processing of His-Prot-Pol*, we already suspected that the epitopes of the Mabs from the 55B20 group were located within the protease moiety, while the epitopes of the 55A10 group were located within the RdRp moiety. To unambiguously assign the epitopes of our Mabs to the mature DWV protein species, we truncated His-Prot-Pol* and generated an N-terminal His-tagged 3CL protease (His-Prot, G_2119_-Q_2393_, calculated molecular mass: 32 kDa). The proteins of induced cultures of *E. coli* expressing either the His-Prot-Pol* immunogen or only the mature 3CL protease (His-Prot) were resolved via SDS-PAGE, blotted onto nitrocellulose membranes, and probed with our new Mabs.

**Figure 10:**
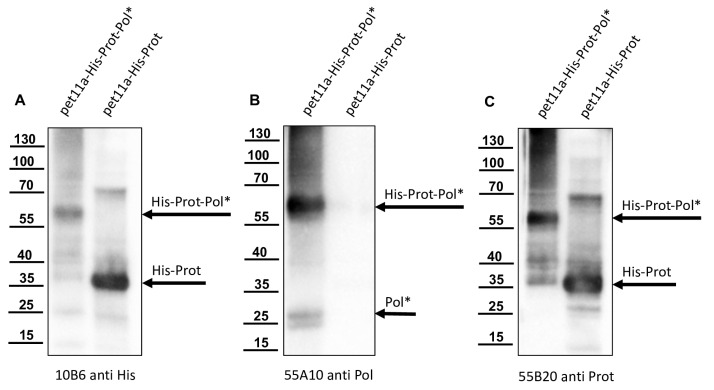
Mab 55B20 is reactive against the mature 3CL protease (His-Prot), while Mab 55A10 is reactive against the N-terminus of 3DL. Total protein of induced cultures of *E. coli* transformed with plasmid pet11a-His-Prot-Pol* and pet11a-His-Prot (G_2119_-Q_2393_) were resolved in SDS-PAGE and analyzed via Western blot, side by side. (**A**) Mab 10B6 anti-His showed only the full-length His-Prot-Pol* (60 kDa) and the His-Prot (35 kDa). Note the weaker expression of His-Prot-Pol* in direct comparison to the His-Prot. Because of the shorter exposure time of this image, the cleavage product His-Prot is not visible in the case of pet11a-His-Prot-Pol*. (**B**) Mab 55A10 anti-Pol reacted with the 60 kDa Prot-Pol*-His and the smaller fragments of 26 and 28 kDa representing released Pol*-His. No reactivity of Mab 55A10 was seen against proteins in the culture expressing pet11a-His-Prot. (**C**) Mab 55B20 anti-Prot showed bands at 60 kDa in the case of His-Prot-Pol* and of 35 kDa for the mature protease in the case of His-Prot. Taken together, it can be clearly seen that Mab 55A10 targets the polymerase region and Mab 55B20 targets the protease region.

An anti-His Western blot (Figure 10A) was used for comparison ensuring correct expression of proteins, gel loading, and membrane blotting. In this experiment, rather weak signals occurred for the 60 kDa His-Prot-Pol* in direct comparison with the smaller and 32 kDa His-Prot, which yielded very strong signals. An additional reactivity with a protein of about 65 kDa was visible in the His-Prot expression corresponding to a dimer probably formed via the oxidation of thiol groups between two cysteine residues, resulting in a covalent bond. Both Mabs, 55A10 (Figure 10B) and 55B20 (Figure 10C), detected the 60 kDa His-Prot-Pol*, but only Mab 55B20 reacted with the mature 3CL protease of 35 kDa. Therefore, an epitope within the boundaries of the mature 3CL protease (S_2119_-Q_2393_) can be assumed for antibody 55B20. Since antibody 55A10 did not react at all with the histidine-tagged His-Prot construct, it can be concluded that the epitope of this antibody is in the remaining Pol* fragment (S_2394_-K_2630_).

### 3.5. Processing of the 3CL/DL Region of DWV In Vivo

**Figure 11:**
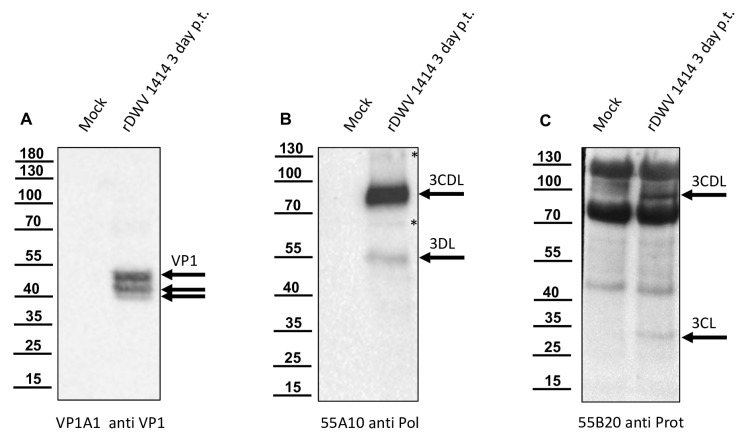
Mab 55A10 and 55B20 detect the 3CL protease/DL as well as mature 3CL protease and 3DL in bee pupae transfected with synthetic DWV RNA. Honey bee pupae transfected with synthetic RNA of the DWV strain 1414 (rDWV 1414) and mock-transfected control pupae were harvested three days post-transfection (p. t.). The pupae were homogenized and total bee protein was resolved via SDS-PAGE. (**A**) Western blot analysis using the anti-VP1 Mab VP1A1. The mock-transfected pupa showed no signal indicating no background infection of the bees. In contrast, a typical VP1 pattern with bands at 47, 42, and 39 kDa appeared in the DWV RNA-transfected pupae, indicating successful infection and viral protein expression. (**B**) Western blot analysis using Mab 55A10 anti-Pol, which targets the 3DL of DWV. The antibody did not show a reactivity against proteins of the DWV-free bee pupae. In the DWV-infected bee pupae, a strong reactivity against a 90 kDa protein and a weak signal indicative of a 55 kDa protein occurred. These reactivities could be assigned to the 3CL protease/DL precursor and the mature 3DL. A 65 kDa band, which cannot yet be assigned more precisely, and a 130 kDa band, which could represent a high-molecular precursor molecule, were marked with an asterisk. (**C**) Western blot analysis using Mab 55B20 anti-Prot, which targets the 3CL protein of DWV. Mab 55B20 showed a strong reactivity with proteins in the uninfected bee pupae, with very strong bands at 75 and 130 kDa and a weaker band at 40 kDa. Despite the strong background reactivities, additional DWV-specific protein bands appeared in the infected pupae. The protein bands at 90 kDa and 33 kDa could be assigned to the 3CL/DL precursor and the mature 3CL protease.

The presence and use of cleavage sites in recombinantly over-expressed polyprotein fragments in *E. coli* provide only evidence for the processing steps and the order of processing realized in the virus-infected cells. Therefore, we applied our new serological reagents for the detection of authentic viral proteins in DWV-infected bee pupae. First, we injected bee pupae with synthetic RNA from our well-established molecular clone of DWV-A, strain 1414 [16]. Pupae of the same age injected with PBS at the same time served as negative controls. After three days of incubation at 33 °C, individual pupae were snap-frozen, homogenized, and lysed in SDS-PAGE buffer. The total bee protein was resolved via SDS-PAGE, blotted onto nitrocellulose membranes, and analyzed. Mab VP1A1 against DWV VP1 was used as an established control reagent verifying DWV infection via the demonstration of VP1 expression [16]. While Mab VP1A1 showed no reactivity with proteins within the lysate of the mock-infected bee pupae (Figure 11A), it showed the three typical bands of DWV VP1 at 47, 42, and 39 kDa in the bee pupae transfected with synthetic DWV RNA. The protein bands represent mature VP1 (calculated molecular mass: 46.6 kDa) and two smaller VP1 fragments that have not been characterized in detail yet.

Mab 55A10 directed against the RdRp region of DWV showed no reactivity with proteins in the lysate of the mock-infected pupa (Figure 11B). This Mab had a very strong reactivity with a 90 kDa protein and weak reactivity with a 55 kDa protein in the lysate of the DWV-positive pupa. Other very weak signals occurred at 65 and 130 kDa, which contrasted only weakly with the background. The major protein band at 90 kDa corresponds to the protease-polymerase precursor (3CDL, calculated molecular mass of 87.2 kDa). The 55 kDa band would fit a mature RdRp (3DL, calculated molecular weight of 56.4 kDa). The 65 kDa band cannot be assigned more precisely so far, because in addition to further cleavage sites within the 3CL protease, processing within the RdRp might also occur, as well as post-translational modifications. The same applies to the 130 kDa band, which might represent a precursor molecule.

Mab 55B20, targeting the 3CL protease, showed very strong reactivities with honey bee proteins in the uninfected bee pupae, which were particularly pronounced at 75 and 130 kDa (Figure 11C). Since other different 3CL protease-specific Mabs obtained from the fusion experiments also recognized the 70 and 130 kDa bands, these Mabs possibly all represent similar IgG molecules originating from one B-cell clone. However, despite their strong background reactivities, DWV-specific proteins could be detected via Mab 55B20, with rather weak bands at 90 kDa and 33 kDa. While it can be assumed that the 33 kDa band represents the mature 3CL protease (calculated molecular mass: 30.8 kDa), the 3CDL precursor was already characterized by the binding of Mabs 55A10 (see above). In summary, during replication of DWV in honey bee cells, the 3CDL region is cleaved at two sites. 3CDL protease seems to be cleaved from the polyprotein efficiently, since no clear bands of larger precursors were visible. In contrast, a slow processing between the 3CL protease and the 3DL RdRp results in minor bands of the mature proteins. The size of the cleavage products was consistent with the sequences of the mature proteins identified via our recombinant expression system.

### 3.6. The Mutations Q_2393_A and Q_2118_A at the Cleavage Sites Prevent the Autocatalytic Cleavage of Recombinant Proteins

**Figure 12:**
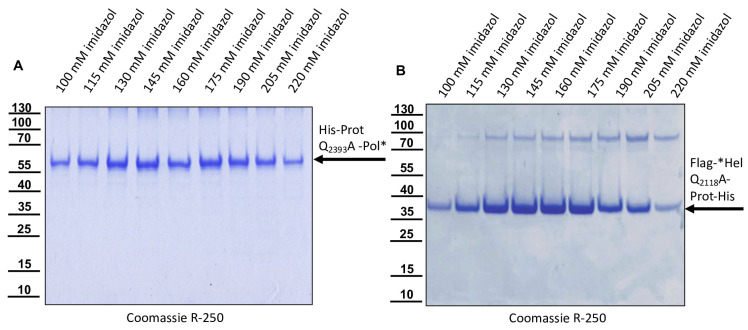
No cleavage products could be purified from the cleavage site mutants ProtQ_2393_A-Pol*-His and Flag-*HelQ_2118_A-Prot-His. (**A**) The insoluble fraction of a lysate from induced cultures of *E. coli* transformed with the plasmid pet11a-ProtQ_2393_A-Pol*-His was solubilized in 8 M urea buffer and subjected to IMAC. The individual imidazole elution fractions were resolved via SDS-PAGE and stained. Note the single protein species with an apparent molecular mass of 60 kDa (ProtQ2393A-Pol*-His) that specifically eluted with increasing imidazole concentrations and the absence of a 28 kDa band (Pol*-His). (**B**) The insoluble fraction of a lysate from induced cultures of *E. coli* transformed with the plasmid pet11a-Flag-*HelQ_2118_A-Prot-His was solubilized in 8 M urea buffer and subjected to IMAC. The individual imidazole elution fractions were resolved via SDS-PAGE and stained. Note the single protein species with an apparent molecular mass of 38 kDa (Flag-*HelQ_2118_A-Prot-His) that specifically eluted with increasing imidazole concentrations and the absence of a 35 kDa band (Prot-His).

The glutamine at position 2393 was exchanged against an alanine (Q_2393_A) to destroy the determined cleavage site between the protease and the polymerase in the recombinant Prot-Pol*-His. The protein was expressed in *E. coli* from plasmid pet11a-ProtQ_2393_A-Pol*-His and purified via IMAC to demonstrate that cleavage was prevented by destruction of the cleavage site. As expected, only the uncleaved ProtQ_2393_A-Pol*-His protein with an apparent molecular mass of 60 kDa was found in the elution fractions, but no cleaved Pol*-His fraction of 28 kDa was detected (Figure 12A). Similarly, the glutamines at position 2118 were exchanged against an alanine (Q_2118_A) to destroy the determined cleavage site between the helicase and the protease in the recombinant Flag-*Hel-Prot-His. The protein was expressed in *E. coli* from plasmid pet11a-Flag-*HelQ_2118_A-Prot-His and purified via IMAC to demonstrate that cleavage was prevented by destruction of the cleavage site. Again, only the uncleaved protein (Flag-*HelQ_2118_A-Prot-His) with an apparent molecular mass of 38 kDa was found in the elution fractions, and the processing product Prot-His with 35 kDa was not detected (Figure 12B). We concluded that in both cases, a P1 residue essential for cleavage had been mutated, inhibiting autocatalysis.

### 3.7. The 3CL-Mediated Processing of the 3C/D Region Is Essential for DWV Replication

**Figure 13:**
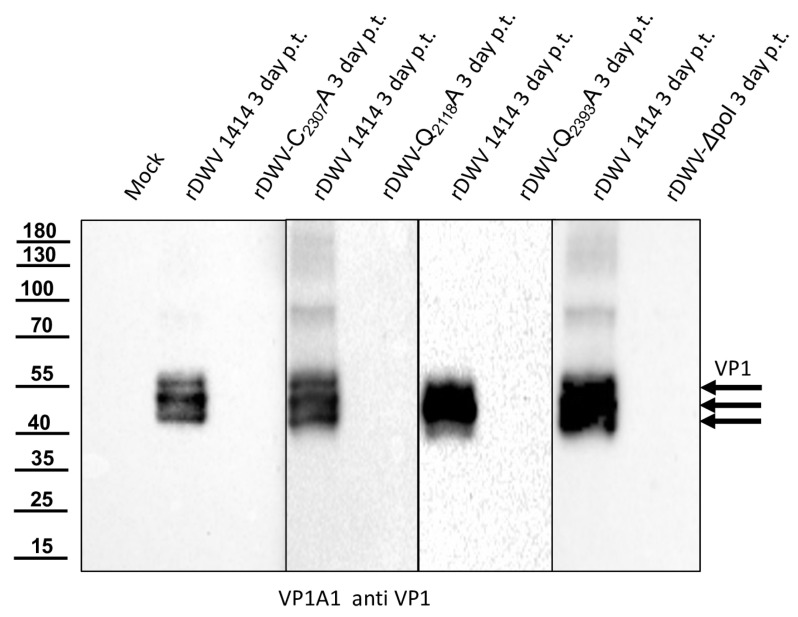
Western blot analysis of 3CL protease and cleavage site mutants. Honey bee pupae were transfected with synthetic RNA of DWV strain 1414 (rDWV 1414), a mutant genome of DWV with an exchange of the active cysteine (rDWV-C_2307_A), a mutant genome with an exchange of the 3BCL cleavage site (rDWV-Q_2118_A), a mutant genome with an exchange of the 3CDL cleavage site (rDWV-Q_2393_A), and a mutant genome with a deletion of the complete 3DL (rDWV-Δpol). DWV RNA transfected and mock-transfected control pupae were harvested three days post-transfection (p. t.). The pupae were homogenized and total bee protein was resolved via SDS-PAGE. A Western blot analysis of the honey bees using anti-VP1 Mab VP1A1 showed no signal in the mock-transfected pupa, and no signals in the bee pupae transfected with DWV genomes with mutations of the active cysteine, the N-terminal, and the C-terminal cleavage site of 3CL protease or with a deletion of the complete RdRp gene. In contrast, a typical VP1 pattern with bands at 47, 42, and 39 kDa appeared in all wild-type DWV RNA-transfected pupa indicating successful transfection, infection, and virus growth. Because the transfections and mutation analyses were performed at different time points and analyzed on different Western blots, the signals from the wild-type control infections are included side by side for each analysis to control RNA synthesis, transfection and VP1 signal intensity, respectively.

To further investigate the importance of the identity of the individual cleavage sites at the N- and C-terminus of the authentic 3CL protease, we generated three DWV mutants, in which either the glutamine cleavage site residues at position 2118 (rDWV-Q_2118_A) or 2393 (rDWV-Q_2393_A) or the active cysteine residue within the protease at position 2307 (rDWV-C_2307_A) were replaced by an alanine. In vitro transcribed synthetic RNA of the mutant genomes was injected into bee pupae side by side with synthetic RNA of our wild-type genome. Again, PBS-injected bee pupae were used as mock controls. The bees were harvested by freezing, homogenized, and lysed 72 h after RNA injection. The total bee lysate was resolved via SDS-PAGE and probed in Western blots using Mab VP1A1 directed against the VP1 (Figure 13). Honey bees injected with PBS showed no signal after probing with the anti-VP1 Mab (VP1A1). As expected, the bee pupae injected with the wild-type DWV genome showed strong signals for the VP1 at 47, 42, and 39 kDa indicating establishment of DWV infection. Neither RNA transcribed from rDWV-C_2307_A nor RNA transcribed from rDWV-Q_2118_A or rDWV-Q_2393_A showed a VP1 signal in our Western blot analyses indicating no or very weak virus growth. An additional negative control RNA lacking the complete 3D sequence (rDWV-Δ3D) served as a control for background protein expression from the transfected synthetic RNA. Since the mutant DWV genomes were tested in an animal infection model, it is difficult to determine which step of the infection cycle is defective. In such an assay, the full infection cycle must run efficiently to cause a systemic infection with detectable amounts of protein production. Therefore, the lack of detection of VP1 expression solely suggests that replication of DWV RNA and/or production of infectious virions is disturbed significantly by each of these mutations.

### 3.8. The 3CL Protease-Mediated Processing of the 3C/D Region Is Essential for DWV Replication

**Figure 14:**
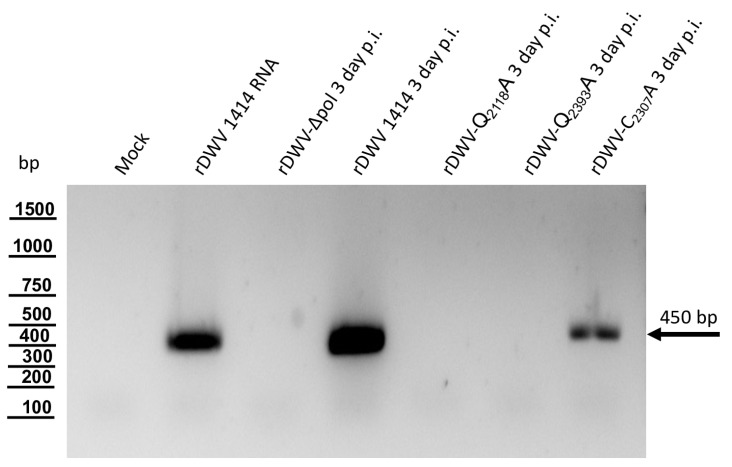
RT-PCR analysis of 3CL cleavage site mutants. Honey bee pupae transfected with synthetic RNA from DWV strain 1414 (rDWV 1414), a mutant genome of DWV with an exchange of the active cysteine within the 3CL protease (rDWV-C_2307_A), a mutant genome with an exchange of the 3BCL cleavage site (rDWV-Q_2118_A), a mutant genome with an exchange of the 3CDL cleavage site (rDWV-Q_2393_A), and a mutant genome with a deletion of the complete 3DL (rDWV-Δpol) were analyzed in RT-PCR. DWV RNA-transfected and mock-transfected control pupae were harvested three days post-transfection (p. t.). The pupae were homogenized, free synthetic RNA was digested with benzonase and total digestion protected RNA was prepared. A 450 bp amplicon of the DWV genome was amplified in a 40-cycles RT-PCR end-point assay. No amplification product became visible in the mock-transfected pupa, the N-terminal and the C-terminal 3CL cleavage site mutants and in the case of the mutant genome with a deletion of the RdRp gene. In contrast, the specific amplicon with a length of 450 bps appeared in the RT-PCR positive control and in the wild-type DWV genome-transfected pupa. Surprisingly, we also detected a weaker band of 450 bps in the 3CL cysteine mutant rDWV-C_2307_A indicating residual genome replication and/or RNA encapsidation.

We also analyzed RNA replication of the 3CL cleavage site mutants, using an established RT-PCR assay to support our conclusions. We included a benzonase treatment of the bee lysates preserving only encapsidated or otherwise protected RNA to prevent the detection of the transfected synthetic RNA. Again, three days after injection of our mutant genomes and control RNAs, the bee pupae were harvested and frozen. After homogenization of the pupae, we digested free, non-encapsidated RNA with benzonase eliminating background signals of remaining synthetic RNA molecules or DNA contaminants from the RNA transcription reaction. Then, total RNA was prepared from the digested samples. We tested 1 µg of the RNA preparations in a DWV-specific RT-PCR assay, which had been presented earlier [16]. While no RT-PCR product was obtained from our mock control pupae, we specifically amplified a DNA fragment of 450 bp from the pupae, which had been injected with DWV wild-type RNA (rDWVwt) (Figure 14). Injection of the in vitro transcribed RNA of the truncated genome rDWV-Δ3D did not result in detectable amounts of RT-PCR products, indicating that the benzonase digest successfully removed all synthetic RNA remnants and/or DNA contaminants. Also, no RT-PCR product was obtained from pupae injected with RNA from rDWV-Q_2118_A and rDWV-Q_2393_A. Since production and/or encapsidation of RNA was below the limit of detection of our assay, we concluded that the identity of the cleavage sites residues Q_2118_ and Q_2393_ was essential for RNA replication and/or encapsidation and protection against benzonase treatment. Surprisingly, however, a weaker signal representing the specific DWV amplicon was detectable in samples from bees that had been injected with synthetic RNA of rDWV-C_2307_A. This result was reproduced in the other bees transfected with rDWV-C_2307_A. Passage of these bee lysates using naive pupae resulted in no first-passage detection of DWV RNA or proteins, suggesting that no reversion of the cysteine mutation in rDWV-C_2307_A had occurred. We concluded that weaker RNA replication and/or encapsidation still occurred in the case of rDWV-C_2307_A. However, Western blot analyses showed no detectable expression of VP1 in rDWV-C_2307_A, and no generation of infectivity was detected in the passage experiment. In summary, it can be deduced from the experiments that Q_2118_ and Q_2393_ represent critical amino acid residues for the replication and encapsidation of DWV RNAs.

### 3.9. The Identity of Residues H_2190_, D_2225_ and D_2199_ Is Not Essential for Replication and Growth of the DWV, Whereas the Mutation of C_2307_, H_2170_ and N_2227_ to Alanine Is Not Tolerated

**Figure 15:**
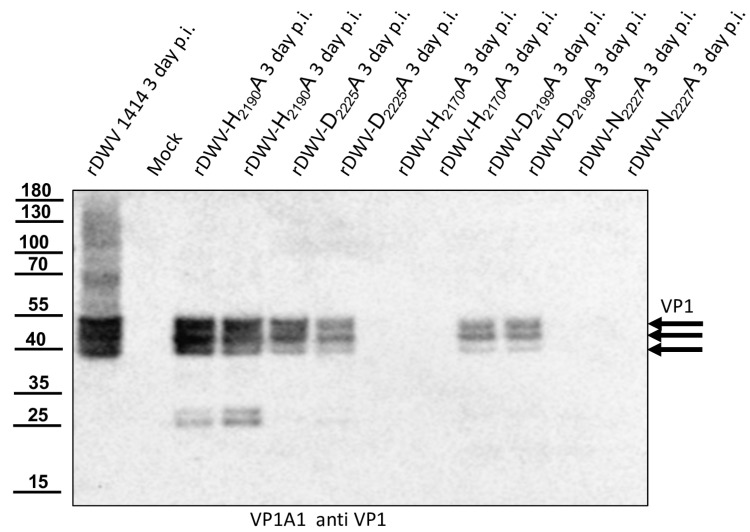
Western blot analysis of DWV mutants with an alanine exchange of residues potentially involved in 3CL catalysis. Honey bee pupae were transfected with synthetic RNA of DWV strain 1414 (rDWV 1414) and mutant genomes containing the amino acid exchanges H_2190_A, D_2225_A, H_2170_A, D_2199_A, and N_2227_A. Transfected and mock-transfected control pupae were harvested three days post-transfection (p. t.). The pupae were homogenized, and total bee protein was resolved via SDS-PAGE. A Western blot analysis using the anti-VP1 Mab VP1A1 showed no signal in the mock-transfected pupa and a typical VP1 pattern with bands at 47, 42, and 39 kDa in the wild-type DWV RNA-transfected pupa. The exchange of H_2190_, D_2225_, and D_2199_ against an alanine was tolerated by DWV in terms of RNA replication, virus growth, systemic infection, and detectable VP1 expression. However, rDWV-H_2190_A and also partially rDWV-D_2225_A showed an aberrant VP1 pattern with smaller bands at 28 and 25 kDa. In addition, rDWV-H_2190_A, rDWV-D_2225_A, and rDWV-D_2199_A showed weaker VP1 signals, indicating compromised viral growth. The mutations H_2170_A and N_2227_A were not tolerated by DWV, and no VP1 signal could be detected after transfection. The experiment demonstrates the importance of the identity of these two residues for 3CL function. The results of two different RNA syntheses and independent infection experiments are shown side by side in the analysis to allow for a more precise comparison to rDWV 1414 (wild-type) protein expression.

Recent data on the in vitro activity of the 3CL protease of DWV suggest that, in addition to C_2307_ and H_2170_, not one of the aspartates (residues 2199 or 2225), but N_2227_ forms the active site of the enzyme. Having already demonstrated the importance of the cleavage sites and the active C_2307_, we also wanted to test the other residues in vivo in the context of a replicative virus system. Because we were still able to detect viral RNA via RT-PCR after mutation of the central catalytic cysteine residue, which is essential for virus growth (C_2307_) and propagation, we opted for Western blot analyses, which demonstrated the importance of C_2307_ clearly. After transfection of rDWV-H_2190_A, -D_2225_A, -D_2199_A, -C_2307_A, -H_2170_A, -N_2227_A and recombinant wild-type RNA (rDWV) and incubation for three days, bee pupae were harvested, snap-frozen, and lysed in SDS-PAGE buffer. In Figure 15, we show two individual transfected bee pupae for each mutant to demonstrate the consistency of different processing patterns and protein expression levels of these protease mutants. The Western blot against the VP1 protein demonstrated the characteristic pattern of VP1 in the wild-type virus RNA-transfected bees, with bands at 47, 42, and 39 kDa, and showed no bands in the mock (PBS)-transfected bees (Figure 15). The mutant rDWV-H_2190_A showed a wild-type-like pattern of VP1 but with additional bands at 24 and 26 kDa, which might represent non-specific cleavage or protein degradation products. The mutant rDWV-D_2225_A showed a weaker expression of the characteristic VP1 bands and additional weak bands at 24 and very weak at 26 kDa. The mutant rDWV-H_2170_A showed no protein expression at all. The mutant rDWV-D_2199_A showed a weak but typical expression pattern. The mutant rDWV-N_2227_A showed no protein expression at all. Taken together, it is obvious that the identity of residues H_2190_, D_2225,_ and D_2199_ is not essential for the replication and growth of DWV, since genomes harbouring an alanine exchange of these residues were able to cause systemic DWV infections in the host. In contrast, residues C_2307_, H_2170_, and N_2227_ are crucial for DWV replication and growth, indicating a direct involvement in catalytic mechanism of the 3CL protease or an essential role for the formation of the backbone structure of the enzyme.

## 4. Discussion

The individual virus species within the order *Picornavirales* are very diverse and grouped in eight separate families. While members of the families *Caliciviridae*, *Picornaviridae*, *Secoviridae*, *Polycipiviridae*, *Solinviviridae*, and *Marnaviridae* infect vertebrates, plants, or algae, the ifla-, dicistro-, polycipi- and solinviviruses (families *Iflaviridae*, *Dicistroviridae*, *Polycipiviridae*, and *Solinviviridae*) exclusively infect invertebrate hosts. Despite their different host range and remarkable sequence divergence, the members of the order *Picornavirales* exhibit many similarities. Common to them all, for example, is a proteolytically processed polyprotein including a block of replicase enzymes consisting of a superfamily III helicase, a proteinase with a chymotrypsin-like structure and a superfamily I RdRp [23]. The viral polyprotein undergoes a complex processing during and after IRES-mediated translation. First, larger blocks of functionally related proteins (P1, P2, and P3 block) are released, which subsequently give rise to the individual mature proteins. However, a spectrum of different types of processing is observed in the different families and genera, which are related to the different translational strategies and differences in the genome structures [44].

A well-studied and typical example for these processes is the enterovirus polyprotein. In enteroviruses, a viral autoprotease (2A) splits off the P1 block already in translation due to ribosomal skipping of the peptide bond releasing the structural protein region from the nascent remaining polyprotein (P2 and P3) [44]. Subsequent or secondary cleavages are mediated by the activity of the main protease (3C) cleaving between the mature viral structural proteins, except for the VP0 cleavage (between VP2 and VP4). In the non-structural proteins, a complex series of events starts with the release of the 3C protease or, more precisely, with the release of its precursors (3CD) via cis-acting auto-proteolysis at the 3B-3C bond. Further trans-cleavage sites at the 2B-2C and 2C-3A bonds are highly sensitive to 3C-mediated catalysis and are efficiently cut. Therefore, an intact P2-P3 block precursor is rarely seen. In contrast, the rather stable 2BC from the P2 block and the 3AB and 3CD precursors from the P3 block are only inefficiently converted into the mature proteins, so that these precursors dominate in infected cells. At least in the case of cleavage of the 3C-3D bond, it can be assumed that cleavages occur mainly as autocatalytic cis-events, since no dilution effects on cleavage have been observed in recombinant purified 3CD. The 3A-3B bond is very stable and is likely to be cleaved only following specific protein–RNA interactions that lead to covalent binding of the 3B to the 5’-end of the genome (viral protein genome-linked; VPg). For the protease, basically four 3C-containing protein species (intact P3 block, 3ABC, 3CD, and mature 3C) can be found in the infected cells. However, 3CD and mature 3C are the most abundant and the other proteins are hardly detectable. In the case of the RdRp, larger amounts of the 3CD precursor and mature protein 3D are found in the infected cells, while only traces of the complete P3 block are detected. All of the 3C precursor molecules, such as 3CD, have an active protease that can catalyze the cleavages between the structural proteins [45].

The most common naturally occurring cleavage sites of 3C proteases as P1-P1’ are Q-G, Q-S, and E-S, although Q-A, Q-C, or even Q-T and Q-I sequences are also cleaved in some cases. Both the different forms of the 3C proteases and the different sequences of the cleavage sites, appear to play regulatory roles. The same is true for the homologous 3C-like proteases of the other members of the order Picornavirales, which were named 3CL since the name 3C was reserved exclusively for the true picornaviruses (sensu stricto family *Picornaviridae*).

Although there are many exceptions, it can be stated that the 3CL proteases prefer (alpha-)glutaminyl residues at the P1 position (E and Q) and small neutral (G and S) or small hydrophobic amino acids (A) at the P1’ position [46,47]. A general sensitivity to thiol inhibitors such as NEM, Zn+, or iodoacetamide as well as a catalytic triad consisting of histidine, aspartate, and cysteine are, with minor exceptions, further common features among the 3CL proteases. Therefore, the 3C/3CL proteases are also classified together in the C3 peptidase family, which is therefore also called the picornain protease family, within clan PA (PROSITE entry PS51874, https://prosite.expasy.org, accessed on 1 October 2023).

The processing of the nonstructural protein region and the responsible 3CL protease of members of the family *Iflaviridae* have not been studied in much detail so far. However, several studies have investigated the enzymatic properties of the 3CL protease of various iflaviruses and have also begun to determine the boundaries of the mature 3CL protein. Ye and colleagues compared the 3CL sequence of the known iflaviruses [26,48,49,50,51,52] back in 2012 and identified a potential catalytic triad of H_2261_, D_2299_, and C_2383_ in the Eov (amino acid numbers refer to the Ectropis obliqua virus, Genbank entry AY365064), which could be substantiated via mutational analyses in a bacterial expression system [37]. However, the role of D_2299_ could only be partially confirmed, as residual activity of the Eov 3CL-D_2299_A was still observed. In the same study, the N- and C-termini of the 3CL protease of Eov were identified as A_2193_ and Q_2491_ using Edmann degradation revealing P1-P1’ cleavage site sequences of E-A and Q-S. A very comprehensive paper on DWV’s 3CL protease was published by Xuye Yuan and his colleague Tatsuhiko Kadowaki. They determined the N-terminus of DWV 3CL protease experimentally as G_2119_ using a bacterial expression system and mutational analyses. Self-cleavage of a glutathione S-transferase (GST) 3CL fusion protein (GST-3CL) could be completely prevented if the cleavage site P1 residue Q_2118_ was replaced with an alanine [40]. The cleavage at the G_2119_ residue and the corresponding Q-G cleavage site have been already predicted by Lanzi et al. in the initial description of the DWV genome [26]. Furthermore, Yuan et al. used AlphaFold2 to model the structure of the 3CL protease of DWV and of many other insect- and iflaviruses, uncovering invariant cysteines and histidines as well as an unusual variety of different amino acids with an oxygen in the side chain as a third residue of the catalytic triads. Alanine substitution and enzyme studies provided additional evidence for an active center of the DWV 3CL protease containing the amino acids H_2170_, C_2307_, and N_2227_, which were all pivotal for the enzymatic activity. A residual activity of 50% was observed if the conserved D_2225_ was replaced by an alanine. Using affinity-purified rabbit antisera [53], they detected the 3DL of DWV in infected bee pupal cells in Western blots as a 55 kDa protein and the 3CDL precursor as a 97 kDa protein. Furthermore, they generated a 3CL-specific rabbit antiserum, which showed a 42 kDa 3CL protease together with the 97 kDa 3CDL precursor. In another very recent publication, Palmer-Young and colleagues used a similar system to investigate the temperature sensitivity of DVW 3CL protease in relation to the honey bee’s life and honey bee’s temperature tolerance [41].

In this study, we aimed to elucidate the processing of the 3CL and 3DL region of DWV in more detail. We produced a 3CL-3DL polyprotein fragment in *E. coli* and purified it via IMAC. This recombinant polyprotein fragment has been successfully used to generate mouse monoclonal antibodies that bind to epitopes in both 3CL and 3DL. Mab 55A10 anti-DWV detected a 3CDL precursor of 90 kDa as well as the mature 3DL with 55 kDa in Western blot analyses of DWV-infected bee pupae. Mab 55B20 anti-DWV detected the 3CDL precursor of 90 kDa and a mature DWV 3CL protease of about 32 kDa. The apparent molecular masses shown by our new monoclonal antibodies, obtained using a tricine-SDS-PAGE system, fit well with the experimentally determined boundaries of the proteins (see below). However, the pronounced differences between the molecular masses of these proteins with the proteins described by Yuan et al. are puzzling. Yuan et al. found a 3CDL precursor of 97 kDa and a mature 3CL protease with about 42 kDa, which appeared even larger when the size marker of Figure 6C was examined in this publication [40]. However, looking back to the original publication by Wu et al., in which the antiserum used against the RdRp of DWV was first presented, it can be seen that molecular weights of 90 (3CLDL) and 53 kDa (3DL) were reported there [53].

We could show that the N-terminus of the DWV 3CL protease was self-cleaved between Q_2118_ and G_2119_ using Edmann degradation, just as predicted by Lanzi et al. on the basis of sequence comparisons [26] and as confirmed by Yuan et al. using mutational analysis [40]. We also identified the C-terminal residue of the mature 3CL protease by N-terminal sequencing of an RdRp fragment as Q_2393_. To the best of our knowledge, the C-terminus of the DWV 3CL protease, and hence also the N-terminus of the DWV 3DL, have not been characterized before. Our refolded purified polyprotein fragment preparations were stable and not further self-processed during storage or incubation. This suggests that processing of the recombinant proteins occur mainly co-translationally in *E. coli*, as Yuan et al. also observed for their GST-fusion protein [40]. The N- and C-terminal self-cleavages of 3CL protease might represent inefficient, co-translational cis-events in the prokaryotic plasma but seem to be more efficient in the context of virus infection in the authentic polyprotein environment.

Our previous studies on molecular DWV clones have paved the way for additional analyses of the DWV 3CL protease in vivo. We verified our observations using reverse genetics and could demonstrate that 3CL protease activity and the identity of cleavage site residues are pivotal for DWV infection of honey bees. The exchange of the active cysteine (C_2307_) and the exchanges of the two P1 glutamines (Q_2118_ and Q_2393_) against alanine prevented viral protein expression from being detected in the Western blot analysis after transfection of synthetic RNA. No replication or encapsidation of the synthetic input RNA was observed for both cleavage site mutants. Surprisingly, however, low-level replication and encapsidation of the synthetic RNA could be measured after transfection of the rDWV-C_2307_A mutant. The 3CL protease domain of picornavirus-like agents adopts a chymotrypsin-like fold with a cysteine nucleophile in place of a commonly found serine. Serine-to-alanine exchange mutants of various serine protease molecules have already been shown to exhibit a residual activity. Evidence suggests that in these cases, a water molecule could serve as a poor substitute for the serine. Such a water molecule could be equipped with a nucleophilic attack character due to hydrogen bonding of the active histidine [54,55]. This could potentially explain why weak RNA replication still occurred in the rDWV-C_2307_A mutant presented here. Whatever the case, the exchange of the active cysteine 2307 to an alanine resulted in a “non-viable” phenotype, because we could not detect VP1 expression and passage of the virus clone was not successful.

With this result, we decided to also investigate the other potential residues of the catalytic triad of the protease in vivo. Additional essential residues were found in H_2170_ and N_2227_ as postulated by Yuan et al. However, rDWV-H_2190_A and -D_2225_A showed an aberrant processing of VP1 and/or a significant reduction of viral protein expression, which was also observed in rDWV-D_2199_A. It must therefore be concluded that all these amino acid residues have a significance for the activity and/or specificity of the 3CL protease. The active-site residues of 3C proteases are in the cleft between the two barrels with a nucleophilic residue from the C-terminal barrel and a general acid basis with a histidine and glutamate or aspartate residues from the N-terminal barrel. The initial prediction of the catalytic triad of the DWV 3CL protease was carried out by Lanzi et al. solely based on sequence comparison with different ifla- and picornaviruses pointing at triad residues C_2307_, D_2225_, and H_2190_. Yuan et al. recently used the algorithms of AlphaFold2 to predict the protein structure of the 3CL protease of DWV and other insect viruses and found that the side chains of C_2307_, N_2227_, and H_2170_ form the active site of the 3C-like protease of DWV. Bacterial expression constructs with alanine substitutions of individual amino acids revealed that the identity of many amino acids of the 3C-like protease was pivotal for the activity in their cleavage assay. However, Yuan et al. were able to show that H_2190_ did not contribute to catalysis, as the exchange H_2190_A remained without functional effects in their assay. Amino acid D_2225_ was of minor importance, since an alanine exchange only reduced the 3CL activity by half. These analyses are conclusive, and asparagine fits well to a catalytic triad, which can be considered as being analogous to the Ser-His-Asp triad of known serine proteases. Our mutational analyses confirmed these hypotheses in vivo. We were able to inactivate the protease via targeted mutation of the suspected triad residues. However, mutation of other residues, such as D_2225_ and D_2199_, led to an obvious reduction in 3CL activity, restricted replication and reduced virus growth.

Future studies must now clarify to what extent the sequences of the cleavage sites, the protein structures and the cis- or trans-activity of 3CL protease influence the processing and replication of DWV in honey bees. In this study, we were at least able to clarify the boundaries of the 3CL protease beyond doubt, so that future studies can be carried out with the complete enzyme produced in the cell during infection.

## Figures and Tables

**Table 1:** Oligonucleotides used in this study.

Name	Sequence
5′-3C-Prot_fwd	5′-GGATCAACACAACAAGTAGACGCTGCTGTG-3′
Internal-Pol_rev	5′-CTTAGACAACCTTGTTGCCAAATTTGTCCAC-3′
5′-3C-overhang-pet11a_rev	5′-GTCTACTTGTTGTGTTGATCCATGATGATGATGGTGAT-GGTG-3′
Internal-Pol-overhang-pet11a_fwd	5′-GCAACAAGGTTGTCTAAGTAGCTCGAGGATCCGGCTG-CTAAC-3′
NcoI-3′-3C_rev	5′-TTTCCATGGTTGAGCATGAGCTAGCTTTGCATCCACTCTACC 3′
NcoI-Stop-pet11a_fwd	5′-AAACCATGGTAGCTCGAGGATCCGGCTGCTAACA-3′
pet11a-overhang-5′-3C_fwd	5′-AGATATACATATGGGATCAACACAACAAGTAGACGCTG-3′
His-overhang-Internal-Pol _rev	5′-TGATGATGATGCTTAGACAACCTTGTTGCCAAATTTG-TC-3′
Internal-Pol-overhang-His_fwd	5′-TTGTCTAAGCATCATCATCATCATCATCACCACCAC-3′
5′-3C-overhang-pet11a_rev	5′-TGTGTTGATCCCATATGTATATCTCCTTCTTAAAGTTA-AAC-3′
3C-C_2307_A_fwd	5′-GATGGTGTTGCTGGTTCAATATTGTTG-3′
3C-C_2307_A_rev	5′-ACCAGCAACACCATCGCCGTGGTATGG-3′
Internal-Beta-Lactamase_rev	5′-GTCGTTTGGTATGGCTTCATTC-3′
Internal-Beta-lactamase_fwd	5′-GAATGAAGCCATACCAAACGAC-3′
3C-Q_2393_A_fwd	5′-CTCATGCTGCAAGCCCTTCTACTGG-3′
3C-Q_2393_A_rev	5′-TGCAGCATGAGCTAGCTTTGCATCCAC-3′
Flag-Internal-Hel_fwd	5′-GACTACAAAGACGATGACGACAAGGGATTGAAATAT-AGTGAAGCAGT-3′
His-3′-3C_rev	5′-GATGATGTTGAGCATGAGCTAGCTTTGCATCCAC-3′
ATG-Flag-pet11a_rev	5′-CTTGTCGTCATCGTCTTTGTAGTCCAT-3′
3C-overhang-His-tag_fwd	5′-TCATGCTCAACATCATCATCATCATCATCACCA-3′
3C-Q_2118_A_fwd	5′-ACTACTAAGCCTGCCGGATCAACACAACAAGTAGAC-3′
3C-Q_2118_A_rev	5′-GGCAGGCTTAGTAGTAACTGGCAATC-3′
3C-H_2190_A_fwd	5′-AAGTATATTGCTAATCAAGAGACTAGAATG-3′
3C-H_2190_A_rev	5′-CTCTTGATTAGCAATATACTTAAAATA-3′
3C-D_2225_A_fwd	5′-TCATTTGCGAGTAATATCGTGCTTGTGACT-3′
3C-D_2225_A_rev	5′-ATTACTCGCAAATGACTCCTCTCCCGC-3′
3C-H_2170_A_fwd	5′-TTGAGGGCATATATTGAGTCAACT-3′
3C-H_2170_A_rev	5′-AGTTGACTCAATATATGCCCTCAACATTAAACA-3′
3C-D_2199_A_fwd	5′-ATGTCTGGTGCGATTTCTGGTATTGAG-3′
3C-D_2199_A_rev	5′-CTCAATACCAGAAATCGCACCAGACAT-3′
3C-N_2227_A_fwd	5′-TCATTTGATAGTGCGATCGTGCTTGTGACT-3′
3C-N_2227_A_rev	5′-AGTCACAAGCACGATCGCACTATCAAATGA-3′

## Data Availability

All data analyzed or generated during this study are included in the manuscript.
